# Ultrathin Carbon Textures Produced on Machined Surfaces in an Integrated Finishing Process Using Microabrasive Films

**DOI:** 10.3390/ma17143456

**Published:** 2024-07-12

**Authors:** Katarzyna Tandecka, Wojciech Kacalak, Michał Wieczorowski, Krzysztof Rokosz, Patrick Chapon, Thomas G. Mathia

**Affiliations:** 1 Department of Engineering and Informatics Systems, Faculty of Mechanical Engineering and Energy, Koszalin University of Technology, 75-620 Koszalin, Poland; wojciech.kacalak@tu.koszalin.pl; 2 Faculty of Mechanical Engineering, Institute of Applied Mechanics, Poznan University of Technology, 3 Piotrowo St., 60-965 Poznan, Poland; michal.wieczorowski@put.poznan.pl; 3 Faculty of Electronics and Computer Science, Koszalin University of Technology, Sniadeckich 2, 75-453 Koszalin, Poland; rokosz@tu.koszalin.pl; 4 HORIBA Scientific, 14 Boulevard Thomas Gobert, Pass. Jobin-Yvon, 91120 Palaiseau, France; patrick.chapon@horiba.com; 5 Laboratoire de Tribologie et Dynamique des Systemes (LTDS), Ecole Centrale de Lyon, Centre National de la Recherche Scientifique, 69134 Lyon, France

**Keywords:** surface finishing, abrasive film, finishing, superfinishing, carbon, thin carbon layer, carbon texture, micro-finishing

## Abstract

This study presents research into the unique method of depositing carbon layers onto processed surfaces, during finishing with abrasive films, on a global basis. The authors of this article are holders of the patent for this method. What makes this technology outstanding is that it integrates processes, whereby micro-finishing and the deposition of a carbon layer onto freshly exposed surface fragments is achieved simultaneously, in a single process. Among the main advantages accruable from this process is the reduction of surface irregularities, while the deposition of a carbon layer is achieved simultaneously. Ultrathin graphite layers can be widely used in conditions where other methods of reducing the coefficient of friction are not possible, such as in regard to micromechanisms. This article illustrates the application of carbon coating, end on, on a surface processed with abrasive film, containing intergranular spaces, saturated with graphite. Thin carbon layers were obtained on two substrates that did not contain carbon in their initial composition: soda–lime glass and a tin–bronze alloy. It was performed through microscopic examinations of the produced surface, roughness analyses of these surfaces, and analysis of the chemical compositions determined by two methods, namely EDS and GDOES, proving the existence of the coatings. The aim of this paper is to prove the possibility and efficiency of using graphite-impregnated lapping films in the deposition process of carbon films, with improved surface smoothness, durability, and wear resistance. The produced coatings will be tested in regard to their operational properties in further research. The authors underline the potential of this method to revolutionize surface treatment processes, due to the significant advantages it offers across various industries.

## 1. Introduction

There are many technologies for producing carbon layers. The methods of carbon layer formation include many highly advanced techniques. One of these methods is electron-beam partial melting of carbon fibers, used for the formation of high-carbon layers on low-carbon steel, which results in a thickness of 3 mm with 2.2% carbon [1]. Another is the creation of nanostructured Co-containing carbon layers through the dehydrochlorination of polyvinyl chloride in the presence of Co-nitrate, followed by heat treatment, which results in continuous carbon layers [2]. Another is carbon deposition on catalysts; where carbon deposition over a Ni/Al_2_O_3_ methanation catalyst was studied, and it was found that the temperature and H2/CO ratio affect the morphology and amount of carbon deposits [3]. Carbonaceous materials are synthesized with respect to various synthesis techniques, such as chemical vapor deposition, arc discharge, and plasma-enhanced chemical vapor deposition, which differ in terms of the synthesis conditions [4]. Again, it was identified that the geotectonic regime was regular regarding changes in the number of maceral groups under conditions of carbon accumulation. The geotectonic regime also affects the coal properties [5]. The following methods of chemical vapor deposition (CVD) can be used for the creation of carbon layers: chemical vapor deposition includes high temperature CVD, plasma-assisted CVD, metalorganic CVD, laser CVD, hot-filament CVD, and low-temperature CVD [6,7]. Low-temperature CVD is pursued to extend its applications for carbon nanostructure use in the semiconductor industry. The low-temperature CVD growth process involves the catalyst system, a carbon source, a reaction atmosphere, and morphology control with a growth mechanism, such as a vapor–facet–solid (VFS) mechanism. CVD allows one to closely control the high-quality preparation of a few carbon layers over large areas, which therefore facilitates the development of 2D materials from the laboratory into industry [8]. The application of CVD for the deposition of carbon materials includes nanotubes, nanofibers, diamond, graphene, and so on, which relate to applications in the semiconductor industry and microfabrication [9]. Carbon synthesis using the CVD method involves other methods, such as plasma-enhanced CVD, low-pressure CVD, and laser ablation, with different temperatures and pressures. PECVD contributes to carbon layer formation in many ways and has many advantages. Carbon materials, such as diamond-like carbons (DLCs), carbon nanotubes (CNTs), and carbon nanowalls (CNWs), are synthesized using PECVD because of its low-synthesis temperature and its ability to grow vertical free-standing structures [10]. CNW growth depends on the plasma conditions, substrates, temperature, and other parameters; furthermore, a study presents some propositions on how the modification of these parameters can be carried out to get the right film properties [11]. It has already been shown that in PECVD at atmospheric pressure, a uniform layer is formed by balancing oxygen and the precursors, and changing the flow streamlines the substrate stage motion [12]. For in situ plasma diagnosis, optical emission spectroscopy presents the chemical composition and relative concentration of species and the gas temperature, so that you can choose the appropriate deposition parameters for the desired carbon coating. And reactive molecular dynamics simulations demonstrate that plasma-based depositions grow single-walled carbon nanotubes at a low temperature, through ions produced by bombardment and the sputtering of amorphous carbon [13]. Carbon layers obtained by CVD find applications as surface layers on engineering and bi-medical materials, and as nanostructures and carbon nanotubes [14,15]. CVD can deposit quite a few materials like silicon, carbon in the form of nanofibers, nanotubes, diamond and graphene, fluorocarbons, filaments, and tungsten and titanium nitride [9]. Specific applications of carbon layers obtained by CVD include the following: gallium arsenide optoelectronic device structures, spin light-emitting diodes, cutting tools, field emitter devices, heat sinks for electronic equipment, electrode materials, biological sensors, and infrared imaging technology [16,17]. CVD is also used to determine the surface quality of carbon-coated materials, a characteristic which has immense importance in many applications, such as in cylindrical batteries with ternary cathodes. In-situ analysis methods, using scanning probe microscope instruments, have been developed for studying the nucleation and growth of thin film materials obtained by carbon condensation from a hydrogen/methane gas mixture, activated by the “hot-filament” method [18]. All the carbon layer formation methods discussed are separate ones. In this paper, an integrated method using the micro-finishing process is proposed. Here, the integration leads to two processes: a decrease in the roughness of the machined surface and the formation of a carbon texture, whose nature depends on the layout arrangement of the machining marks on the machined surface.

Carbon layers generally do not glue well to substrates and can be improved with the use of a titanium interlayer [19]. They have very good sliding properties, so are great for reducing friction between materials, especially when process fluids cannot be used. Carbon layers are known for their biocompatibility, hardness, abrasion resistance, and high chemical resistance [20,21]. Carbon layers have applications in medicine, machine tools, and as barrier layers, due to their unique properties. They have manifold and tunable properties that make them very suitable for use in flow batteries, as electrodes or bipolar plates [22]. Carbon materials, such as pyrolytic carbon and carbon fibers, in biomedical engineering, have orthopedic and cardiovascular applications, due to their mechanical and biological compatibility within the human body [23,24]. The sliding properties of carbon layers, in combination with their biocompatibility and thermal shock resistance, make them very versatile for many applications across many industries, from mechanics to biomedical engineering. Their physicochemical properties offer several possibilities, making them essential materials that have many uses. There are several types of carbon, with different unique properties. Crystalline carbon, which includes diamond, graphite, and fullerenes, offers benefits such as hardness, electrified thermal conductivity, and anisotropy [25,26,27]. For amorphous carbon, because of the lack of long-range order in the structure, many of its properties are compromised [28]. Carbon nanoparticles have several properties, including high electrical and thermal conductivity, rigidity, and strength [29]. Under many applications in many fields, diamond-like carbon layers play a leading role, with unique physicochemical properties [30]. Carbon nanotubes also exert high electrical and thermal conductivity, rigidity, strength, and hardness, which highlight its many outstanding properties. Lattice defects, thus, bring about non-graphitic carbon, which is associated with broad crystallinity and morphology, and, hence, is associated with its many properties and applications. The molecular structure, hybridization, and bonding are the origins of the properties of these carbon layers [31]. Nevertheless, the terminology and descriptions of carbon materials are tricky due to the many different allotropes of carbon, and a common language is needed among carbon scientists [32]. There are several uses of carbon layers in mechanical engineering, which have been established through various studies. Several uses in mechanics, tissue engineering, and bio-compatibility testing have been found for diamond-like carbon layers. Thin carbon layers offer sliding properties, which are ideal for when process fluids cannot be used, or when the friction between materials needs to be reduced. They are also used in medicine for biocompatibility, in machine tools for hardness and wear resistance, and as barrier layers for high chemical resistance [19]. Carbon-based thin film tool coatings, like diamond-like carbon (DLC), can be applied for cutting and machining of non-metallic and non-ferrous metals, in order to improve their mechanical and thermal properties [33]. Thin layers of different materials are used for protective coatings in order to increase thermal protection, abrasion resistance, oxidation resistance, and corrosion resistance, for electronic packaging, magnetic recording media, and layered structural composites [34]. In this regard, multilayer polyethylenimine/graphene oxide thin films have been shown to exhibit good solid lubrication performance by reducing friction and increasing the wear life of mechanical devices [35]. Thin carbon layers do a lot to aid in the betterment of material properties. Thin carbonic layers, including DLC coatings, are hard, low in friction, and wear resistant; hence, they are good for machine tool applications and as coatings [36]. The doping of metals into carbon films, such as titanium, enhances the adhesion of the carbon layer to the substrate and increases its elasticity, hence offering better mechanical properties [37,38]. Carbon films find applications in medicine because they are biocompatible; thus, they are suitable for use in biomedical implants and tissue culture. In general, multilayer thin carbon films maintain high hardness and enhance friction behavior; hence, they are good for applications where low friction and high wear resistance are needed. Thin carbon layers made from wood or carbon, like wood carbon sponges and carbon nanotubes, are smart materials that show good elasticity and strength retention; hence, they are good for precision engineering industries [39].

**Figure 1:**
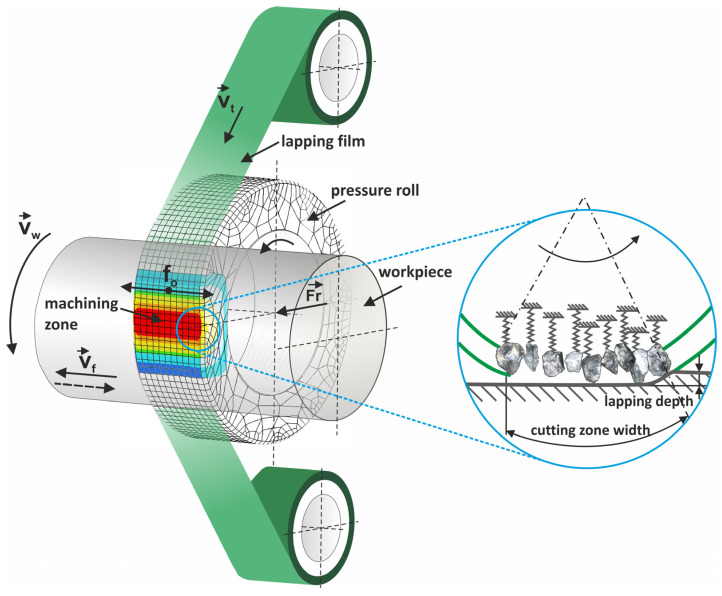
Kinematic diagram of rotary surface superfinishing using lapping films, indicating the following quantities: *v_t_*—tool velocity, *v_w_*—workpiece velocity, *v_f_*—tool feed rate, *f_o_*—tool oscillation rate, and *F_r_*—the pressure force of the pressing roller [43].

Surface micro-finishing processes are characterized by the achievement of an extremely smooth or fine final surface [40]. In the superfinishing process, sequential treatment is conducted, wherein progressively finer grains of microabrasive rolls are used. Normally, the process starts from a grain size of 30 μm, and the nominal grain size can be reduced to as low as 0.5 μm by changing the tool in a sequential manner [41,42]. The abrasive grains that make up the abrasive layer can be made of a variety of materials, for example, noble corundum, synthetic diamond, or silicon carbide [43,44,45,46,47,48]. Notably, this process uses single-use tools. For this reason, it becomes important to optimize the process parameters for the best possible utilization of the tool. The abrasive film moves very slowly, with respect to the feed rate of the workpiece, as shown in Figure 1. Nowadays, this type of treatment is mainly applied to rotating objects, but the technique itself can be used for every shape of processed surface [49].

Carbon layers on workpiece surfaces are of interest because of the possibility of improving the surface properties, cutting down the roughness, and improving the tribological characteristics. This paper deals with a new combined method of superfinishing and deposition of carbon layers, which is a reasonably new approach to technology in that it combines into one process surface smoothing and carbon coating, for which the authors of this article have obtained a patent [50]. The method is significant because it serves the need for more efficient and effective treatment of surfaces in many industries. In the present work, the approach taken is to investigate the feasibility and effectiveness of using graphite-impregnated lapping films in depositing carbon films on soda–lime glass and tin–bronze alloy surfaces. This integrated method is not only useful for reducing surface roughness, but also for improving the durability and wear resistance of treated surfaces. Although studies on individual surface finishing techniques and carbon coating methods have been conducted previously, such a combination of the aforementioned processes has never been undertaken until now. Key papers on surface engineering and tribology indicate that carbon coatings may reduce friction and wear, but the integration of such coatings with the micro-finishing process is an absolutely new approach that may change the concept of surface treatment. The present work proves that a graphite-impregnated lapping film deposits a uniform carbon layer on a machined surface. The process, in other words, simplifies the quality and durability of huge surfaces. Ultrathin graphite layers on smoothed surfaces are useful for systems that remain inactive for a certain period and are required to function efficiently without the need for additional maintenance (systems with limited access to kinematic joints, systems used in military technology, or intended for applications in vacuum and low-temperature environments).

This paper presents research on a new method for the deposition of carbon layers on a processed surface, during the finishing process, with the use of abrasive film. The authors propose micro-finishing techniques combined with the deposition of carbon layers in a single process and underline the novelty of such a process, since this invention is already patented by them. In this way, it is not only possible to reduce surface irregularities, but also to apply a carbon layer onto the surface, which will definitely improve its tribological properties. The obtained ultrathin graphite layers can be applied in a wide range of conditions, when the use of other methods for reducing the friction coefficient is not feasible, for example, in regard to micromechanisms. This research is concerned with the deposition of thin carbon layers on targets like soda–lime glass and a tin–bronze alloy; the quality was checked by microscopic examinations, the assessment of roughness analysis, and chemical composition assessments using EDS and GDOES. This is coupled with the setting of future research directions for testing the operational properties of the produced coatings.

## 2. Materials and Methods

**Figure 2:**
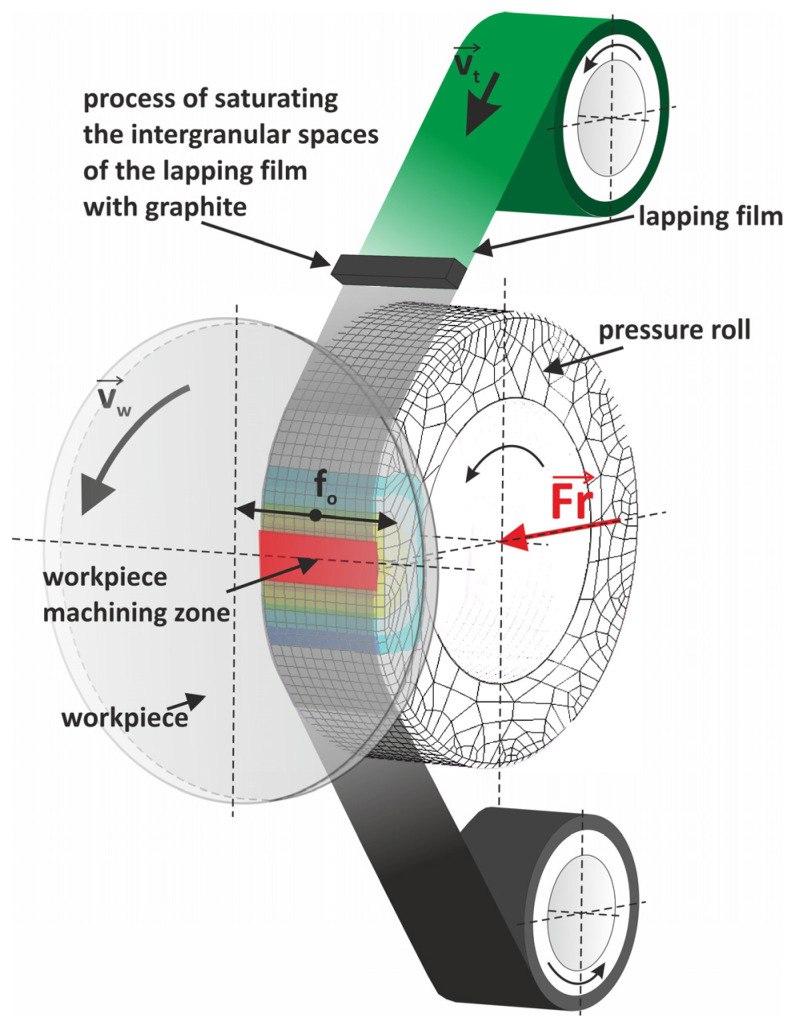
Kinematic diagram of finishing with the simultaneous application of a thin graphite coating on the finished surface, indicating the following quantities: *v_t_*—tool velocity, *v_w_*—workpiece velocity, *f_o_*—tool oscillation rate, and *F_r_*—the pressure force of the pressing roller.

Figure 2 shows the process of applying a carbon layer to the surface of a workpiece using a graphite-impregnated lapping film.

**Figure 3:**
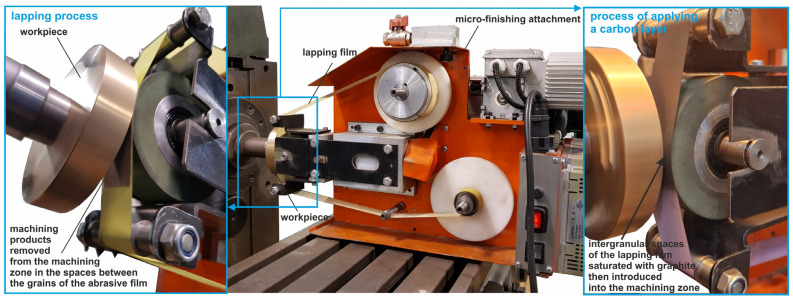
Research setup for the micro-finishing process, with the integrated simultaneous application of thin carbon layers.

The process starts with the lapping film preparation, where the film is saturated with graphite in the intergranular spaces, so the abrasive film carries graphite uniformly. The lapping film is then fed into the system at a certain velocity (*v_t_*). As the lapping film advances, it passes over a pressure roll that applies a certain force (*F_r_*) to the film, which presses it against the workpiece surface. This pressure ensures the graphite is evenly distributed onto the workpiece. The workpiece is positioned in the machining zone and rotates at a certain velocity (*v_w_*), so the entire surface is coated uniformly with graphite from the lapping film. During machining, the abrasive particles in the lapping film lap the workpiece surface and transfer graphite, creating a thin carbon layer. The combination of the pressure roll, the relative motion between the lapping film and the workpiece, and the abrasive nature of the film, results in a polished surface uniformly coated with carbon. A smooth surface and a uniform carbon layer are produced. This process improves the surface properties, reduces roughness, and can improve the tribological properties. This process makes it easy to achieve a fine surface finish with a carbon coating and better performance, for use in many applications. Figure 3 shows the experimental setup for the carbon layer deposition process. The standard machining parameters for the micro-finishing process are presented in Table 1. The machining process was conducted with the GW-1 micro-finishing attachment (Figure 3). This micro-finishing attachment is mounted on an engine lathe in the cutter holder seat and can be used with tools with a width of 1/2”, 1” or 2”. The tool feed rate is *v_f_* = 0–90 (up to 500) mm/min, the oscillation frequency is *f*_0_ = 0–500/min, and the oscillation amplitude is *A* = 2.5 mm. The roll pressure is *F_r_* = 10–90 (up to 200) N, using a pneumatic actuator with a pressure of 0.6 MPa. The attachment is 230 V, 400 W, and 575 × 250 × 300 mm in size, and weighs 25 kg.

**Figure 4:**
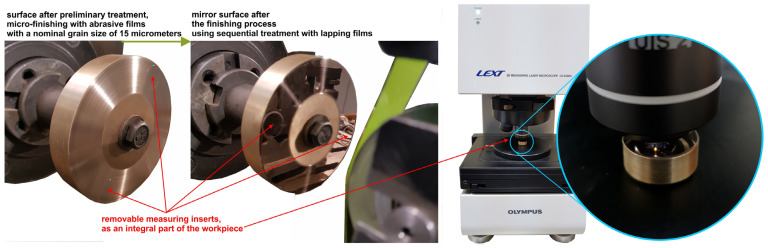
Workpiece before the lapping film process and after the smoothing process, with marked measurement inserts as detachable elements.

The processing of the abrasive film is a sequential operation, which means that the finishing operations are performed using abrasive films with progressively finer grit sizes. The preparatory processing of the bronze alloy for the lapping process involved a micro-finishing process, using a film with a nominal grit size of 15 μm (Figure 4). The lapping process was carried out with the following sequence of abrasive film grit sizes: 30, 12, 9, and 5 μm, for the bronze alloy. For the soda–lime glass, the process was performed in the sequence of 30, 12, 9, 5, 1, and 0.5 μm. The abrasive films with a grit size of 5 μm and larger were made of noble electrocorundum abrasive grains, whereas the films with a grit size of 1 and 0.5 μm were diamond grit films. The workpiece was designed so that its integral part includes removable inserts, allowing unrestricted microscopic examination of the processed surfaces. In each process, two obtained surfaces were compared: one smoothed conventionally and the other with the finest grade film, with the intergranular spaces filled with graphite.

The surface topography measurements of the bronze alloy were taken using the Talysurf CCI 6000 system, from Taylor Hobson in Leicester, England. The measurement field was 899 × 899 μm, with 1024 × 1024 points, and 0.879 μm spacing in terms of X and Y. The Z-axis resolution is 0.001 nm; this is a very precise instrument for surface roughness measurement. The CCI 6000 system has many benefits in terms of measuring smooth surfaces. The SEM and EDS images of the carbon layer surfaces were taken with a Phenom ProX tabletop scanning electron microscope, from Phenom-World BV, Eindhoven, NL. The topography of the soda–lime glass after the machining process was examined using an Olympus LEXT OLS4000 confocal microscope (Tokyo, Japan). Optical images of the produced carbon layers were also obtained using this device. The Glow Discharge Optical Emission Spectroscopy (GDOES) analysis was performed using the Horiba Profiler 2 (Horiba Scientific, Palaiseau, France).

## 3. Results and Discussion

### 3.1. Research on the Carbon Coating Produced on the Surface of Soda–Lime Glass

**Figure 5:**
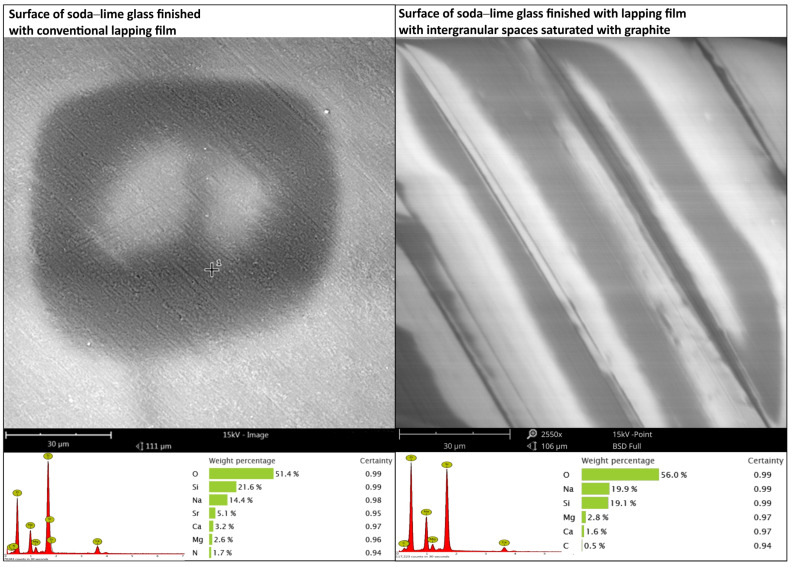
Comparison of soda–lime glass surface finishes: Left: Surface finished with conventional lapping film. Right: Surface finished with lapping film, where intergranular spaces are saturated with graphite. Below: Elemental composition and weight percentages for each surface.

Figure 5 shows the surface of the soda–lime glass with two different treatments: the conventional lapping film and the lapping film with the intergranular spaces filled with graphite. Each image is accompanied by the EDS results for the surface. The lapping film-treated surface looks homogeneous with some darker areas, which are due to the charging effect during the SEM measurement on the non-conductive sample, rather than natural variations in the surface properties or topography. The EDS spectra showed the presence of oxygen (51.4%) and silicon (21.6%) in the soda–lime glass. Notable amounts of sodium (14.4%), strontium (5.1%), calcium (3.2%), magnesium (2.6%), and nitrogen (1.7%) were also present, but these are standard elements found in soda–lime glass. The graphite-saturated lapping film shows parallel lines or bands; thus, it is noted that the occurrence of graphite saturation in the finishing treatment likely created a stratified structure. The EDS test on the composition of this surface showed: 56.0% oxygen, 19.9% sodium, 19.1% silicon, 2.8% magnesium, 1.6% calcium, and small amount of carbon, 0.5%. The bands appear due to the action of graphite in between the surface. This banded structure is the result of the modification to the finishing process caused by graphite, which created this kind of linear feature. The use of conventional lapping film results in a uniform surface, but with some differences, while graphite-saturated lapping film results in a banded structure; during the finishing process, the mechanical action is inhomogeneous. Although the elemental composition, in both cases, is almost the same, the notable difference is the presence of carbon, due to graphite in the lapped sample.

**Figure 6:**
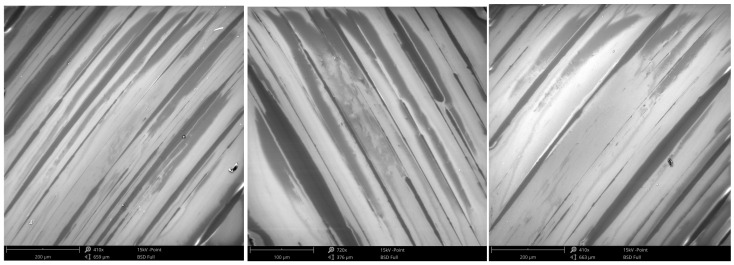
SEM images of the soda–lime glass surface after the micro-finishing process, with a thin carbon layer applied in the form of visible bands.

So, the introduction of graphite into the lapping film changes the surface morphology of the glass, significantly. By analyzing the two SEM images, regular bands are formed on the surface when smoothed with graphite-impregnated film. It may have variations in its mechanical properties, but the elemental composition is almost the same as the soda–lime glass. However, the presence of carbon can be observed, which was not the case for conventionally processed surfaces. After lapping with the graphite-saturated film, the surface shows parallel lines or bands, so the graphite had a big impact on the finishing process and likely formed a layered structure (Figure 6). The carbon layer is very thin, with 0.5% carbon content, as shown in Figure 5. In the spot analysis, which derives part of the elemental composition from deeper within the material, we can still see the charging effect during the SEM measurement, typical for non-conductive samples. The substrate is non-conductive and, interestingly, the charging occurs along the bands on the surface, so the thin layer must have some conductivity. This means that the graphite particles were embedded into the surface during lapping. Graphite not only changes the surface morphology, but also the tribological properties of the glass. The parallel bands may improve the wear resistance by distributing the stress more evenly on the surface. And the thin carbon layer may provide some lubrication, reducing the friction and enhancing the overall durability of the material.

**Figure 7:**
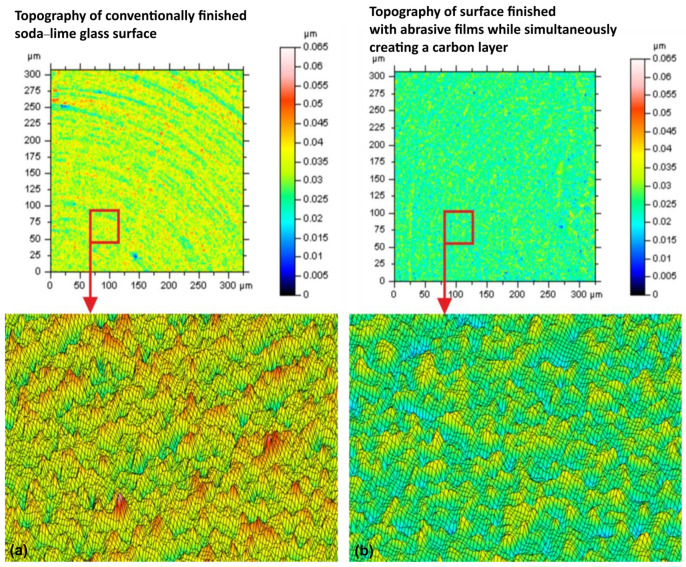
Soda–lime glass topography. (**a**) Conventional soda–lime glass surface. (**b**) Surface finished with abrasive films and carbon layer. Insets: Magnified view of the highlighted area in each topography map.

The topography of the two surfaces as a 2D color map and 3D mesh, before and after the placement of the carbon layer, are shown in Figure 7. The height differences are very noticeable, with much clarity, in the 2D color map. The untreated surface includes a mix of colors, namely green and yellow, which means it has medium roughness, with visible peaks and valleys. The carbon-layer surface is more homogeneous in color, green and blue, which suggests a smoother surface, with fewer height variations. This can also be clearly seen in the 3D mesh views. The untreated surface has big peaks and deep valleys. The color indicates sharp peaks and deep valleys in the 2D color map. Also, the carbon-coated surface is more even, with less peaks and shallower valleys; hence, confirming what can be observed in the 2D color map: that the carbon layer smooths the surface. The analysis of these images reveals a dramatic decrease in the surface roughness due to the carbon layer. Clearly, there is more uniformity in terms of color in the 2D map and a smoother topology in the 3D mesh view. The peak height was reduced and the valley depths were filled out by the carbon layer, thus making the surface more homogeneous. This is beneficial for applications that require less friction and wear; a smoother surface usually performs better under this type of condition. The two surfaces can also be differentiated visually. While the untreated surface is rough with a varied topography, the carbon-coated surface is relatively uniform and smooth. This will be further quantified with respect to the roughness parameters (Sz, Sv, Sp) in the next section. Because the finishing process involves the removal of surface peaks, it first enhances the bearing surface layer through the creation of flat areas. During this process, an undesirable phenomenon occurs, namely that there is greater difficulty in removing individual deep scratches. The coefficient of friction is reduced by the existence of graphite in the machining area, which helps in the minimization of the formation of individual deep machining marks. Specifically, carbon accumulation is most likely in the valleys of the machining marks only, considering the specifics of the machining process. In this way, even when the carbon layer covers the whole surface, it might be partly scoured off during finishing, thus probably forming a carbon texture on the surface.

**Figure 8:**
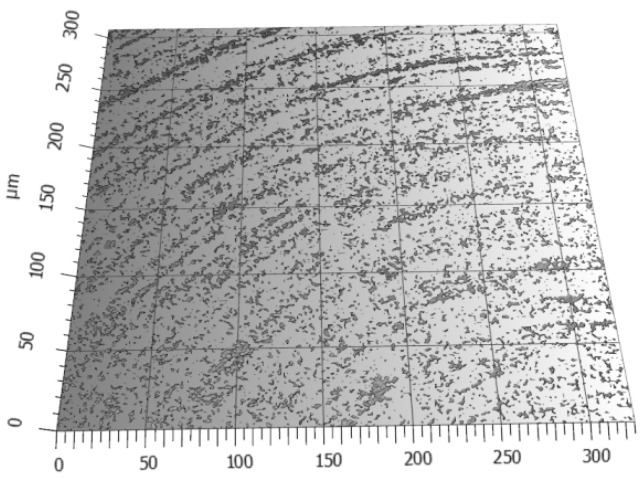
Graphite texture formed on the machined surface. Areas with the highest probability of carbon accumulation in the valleys of machining marks.

In Figure 8, the areas are indicated that have the highest probability of carbon accumulation. This phenomenon of carbon accumulation in these areas is somehow useful for the future operational properties of the surface in regard to two-element interactions, indicated by a lower Sv parameter on the surface.

**Figure 9:**
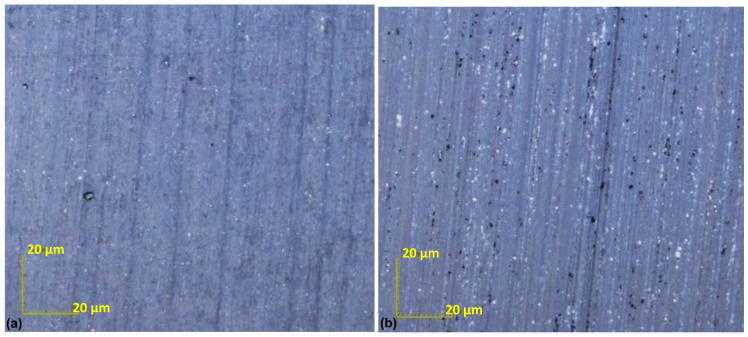
Confocal microscope images in optical mode of the soda–lime glass surface sample after lapping. (**a**) Conventional soda–lime glass surface. (**b**) Surface finished with abrasive films and a carbon layer.

Figure 9 shows the optical images of the two surfaces taken with a Lext OLS 4000 confocal microscope. As they are optical images, we can see that the two surfaces do not have different colors, so the carbon layer is transparent. This means the layer is very thin. The point at which a layer turns colorless is influenced by the material and its optical characteristics. A layer is considered transparent when its thickness is sufficiently small to prevent significant absorption or the scattering of visible light, which has a wavelength ranging from approximately 400 to 700 nanometers.

**Figure 10:**
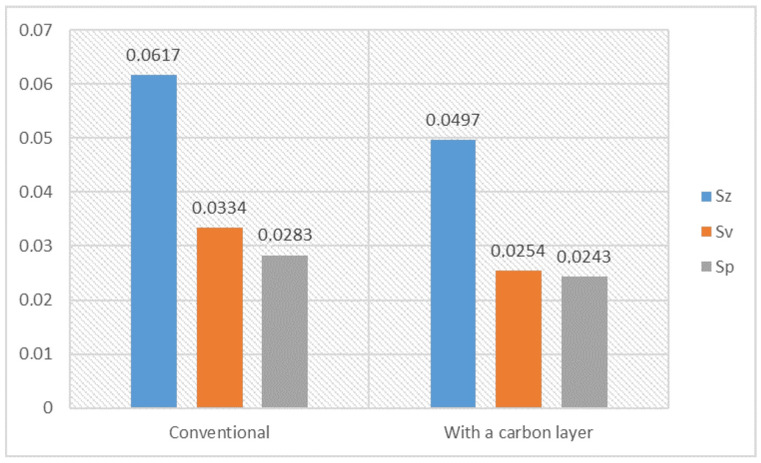
Surface roughness parameters between the conventional surface and the surface with a carbon layer, in micrometers. The parameters measured are the maximum surface height (Sz), maximum valley depth (Sv), and maximum peak height (Sp) in µm.

Below is a comparison of the surface roughness parameters between the conventional surface and the surface with a carbon layer, as illustrated in Figure 10. The parameters measured are the maximum surface height (Sz), maximum valley depth (Sv), and maximum peak height (Sp) [51]. For the conventional surface, the Sz is 0.0617 µm, and for the surface with a carbon layer it is 0.0497 µm. This means the carbon layer smooths out the most significant irregularities on the surface. The Sv of the conventional surface is 0.0334 µm, and for the carbon layer surface it is 0.0254 µm. This means that carbon accumulates in the valleys or machining marks and makes the valleys shallower than the conventional surface. The Sp for the conventional surface is 0.0283 µm, and for the carbon layer surface it is 0.0243 µm. A reduction in the Sp for the carbon layer surface means that the highest peaks are slightly smoothed by the carbon layer, but not as much as the Sz and Sv.

The results of adding a carbon layer on the surface roughness parameters are shown. The addition of a carbon layer reduces the maximum surface height (Sz) and maximum valley depth (Sv), which means that the carbon fills in the valleys created during machining. This results in a smoother surface, with fewer and less pronounced irregularities. A reduction in these parameters means that the carbon layer has a leveling effect, which is good for applications that require a smoother finish and potentially increased wear resistance. While for the Sa parameter, the value equaled 0.00387 μm for the surface without a carbon layer, and with the application of a carbon layer on top, the value diminished to a value equal to approximately 0.0031 μm. From this difference in roughness alone, we could already say with certainty that the friction coefficient would be lower on the surface with the carbon layer. Also, one cannot forget that a carbon layer on a soda–lime glass surface reduces it even more. The relationship between the surface roughness and the coefficient of friction is nonlinear, depending on a lot of factors. For instance, with dry friction, such a change in the surface roughness of soda–lime glass, for simplicity, assuming the coefficient of friction is directly proportional to the surface roughness described by the Sa parameter, can be estimated to reduce the coefficient of friction by 20%. Moreover, the produced carbon layer will decrease the coefficient of friction even more. Carbon, especially graphite, has natural lubricating properties. Graphite consists of layers of carbon atoms, bonded to one another within the layer in a hexagonal structure, making a plane called a graphene plane. Such planes are strongly bonded within the layer because they are of a covalent type, while between the layers, they are much weaker because they are subject to van der Waals forces. This means that upon the application of force, the layers can easily slide on top of one another, resulting in low friction coefficients and, thereby, acting as a lubricant. It is due to the weak interlayer bonds that only a very small amount of energy is required to shift one layer relative to the other in graphite. This easily allows the layer to slide over the other, making the friction less. Graphite is both chemically stable and resistant to high temperatures; hence, it is a very good lubricant under extreme conditions, when most lubricants degrade. It does not generally react with most chemicals, complementing its durability as a lubricant. Graphite has high thermal conductivity, which acts to dissipate the heat generated due to friction. This also contributes to reducing wear and extending the lifespan of contact surfaces. Easily adsorbed graphite layers are likely to form a thin lubricating film on most surfaces. This reduces direct metal-to-metal contact, or other hard material contact, thus further reducing friction and wear. A carbon surface layer can also improve the tribological properties of materials by reducing friction and wear during operation. This will enhance the performance and lifespan of mechanical parts, especially in applications where surface finish and wear resistance are critical. Overall, the use of a carbon layer creates a more uniform and durable surface, which improves the material’s functional properties in practical applications.

**Figure 11:**
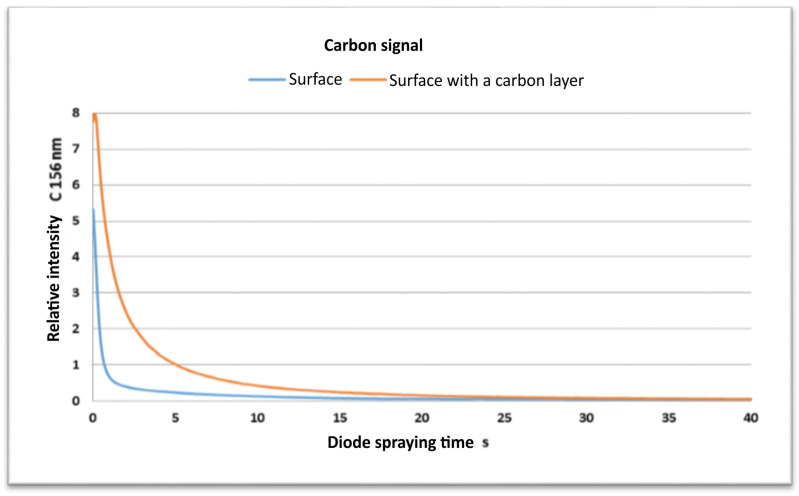
Plot of carbon signal (C 156 nm) versus diode spraying time for surfaces with and without a carbon layer.

Glow Discharge Optical Emission Spectroscopy (GDOES) is a modern analytical technique used to analyze the elemental composition of material surfaces and thin layers. Two samples were measured: soda–lime glass referred to as the “Surface” and soda–lime glass with a thin carbon layer referred to as the “Surface with a Carbon layer”. The objective was to understand how the presence of a carbon layer affects the carbon and oxygen signals during the diode spraying process. Figure 11 shows the relative intensity of the carbon signal (C 156 nm) versus the diode spraying time for both samples. It was found that an inorganic carbon layer is deposited on the sample treated with a lapping film containing carbon. In the initial phase (0–5 s), the carbon signal for the surface with a carbon layer was much higher, about 8 units, compared to the bare soda–lime glass, which was about 1 unit. This means that a thick carbon layer is present on the treated surface of the glass. As the diode spraying process continues, the carbon signal for both samples decreases. For the carbon-coated sample, the signal drops sharply within the first few seconds, meaning that the carbon layer is being removed quickly. The signal for the plain glass also decreases, but at a much slower rate, and converges with the carbon-coated sample at around 15–20 s. The rapid decline in the carbon signal for the treated sample confirms the presence and removal of the carbon layer. The convergence of both signals to near zero means that the complete removal of the carbon layer has occurred, with minimal residual carbon on the glass surface.

**Figure 12:**
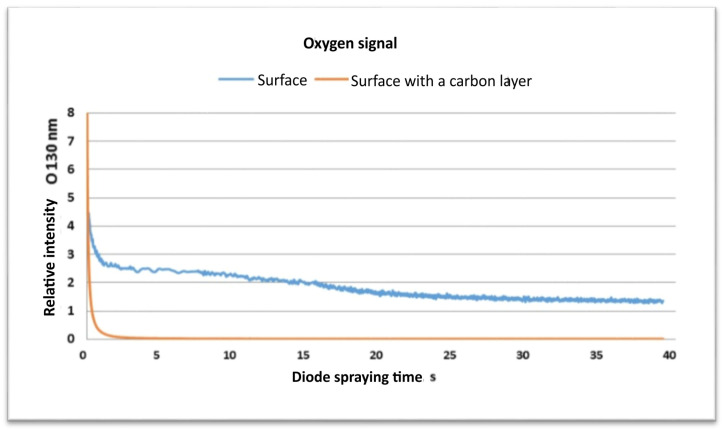
Plot of oxygen signal (O 130 nm) versus diode spraying time for surfaces with and without a carbon layer.

Figure 12 shows the relative intensity of the oxygen signal (O 130 nm) versus the diode spraying time for both samples. Initially the oxygen signal for the plain glass surface is higher, about 4 units, which is the natural oxygen content of soda–lime glass.

For the carbon-covered surface, the oxygen signal is close to zero at the beginning, which means the carbon layer covers the underlying oxygen. As time elapses, the oxygen signal drops for the plain glass, but stays steady, indicating uniform oxygen in the glass matrix. For the carbon-coated sample, the oxygen signal rises as the carbon gets etched away but is still below the level of the plain glass sample. The low initial oxygen signal of the carbon-coated sample indicates that the carbon layer is effective in terms of its coverage of the glass. A stable oxygen signal for the plain glass indicates that there is a complete and uniform distribution of oxygen within the glass. The GDOES analysis provides information on the composition and behavior in relation to the surface of the soda–lime glass with and without a carbon layer. The high initial carbon signal for the treated sample indicates the successful deposition of the carbon layer. A rapid decrease in the signal during diode spraying underlines that the GDOES is effective for removing the surface layer. Further, a low oxygen signature at the beginning of the process, for the carbon-coated sample, indicates that the carbon layer covers the underlying glass composition. The flat nature of the oxygen signal for the plain glass sample shows a uniform elemental composition in the glass matrix. All the results guarantee the power of the GDOES in characterizing surface treatments and thin films on a soda–lime glass substrate.

### 3.2. Research on the Carbon Coating Produced on the Surface of the CuSn7Zn4Pb6/RG7 Tin–Bronze Alloy

**Figure 13:**
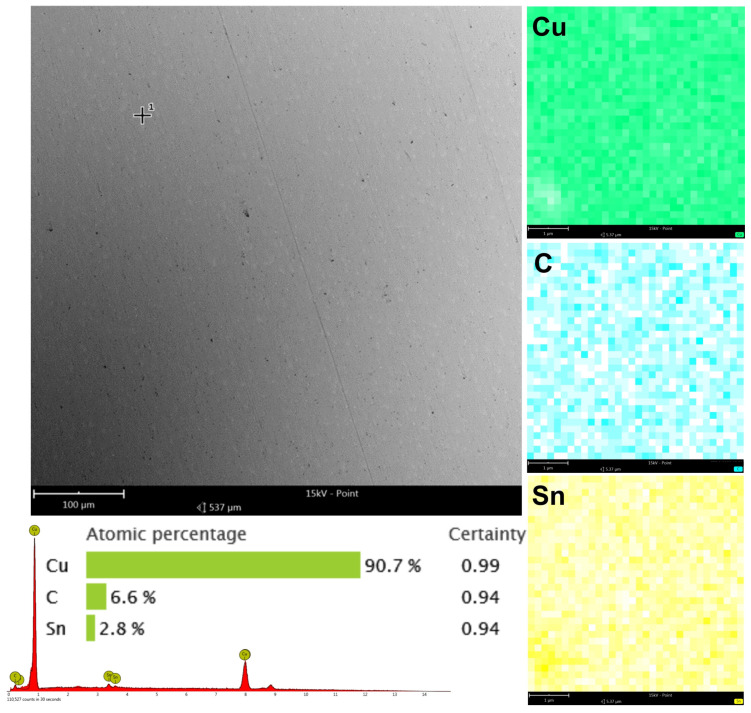
Chemical composition analysis of the surface of the CuSn7Zn4Pb6/RG7 tin–bronze alloy after carbon coating. Top left is the SEM micrograph of the surface, the images on the right are the elemental distribution maps for carbon (C), copper (Cu), and tin (Sn). The bottom chart shows the atomic percentage composition of the detected elements and the measurement certainty: Cu 90.7% (0.99), C 6.6% (0.94), Sn 2.8% (0.94).

EDS is a great way to determine the elemental composition of materials. The EDS results for the bronze alloy with a thin carbon layer are presented (Figure 13).

The point and surface analysis shows the distribution and concentration of elements on the surface. Point analysis shows the atomic percentage of the elements at specific locations in the sample. The detected elements are copper (Cu) 90.7%, carbon (C) 6.6%, and Tin (Sn) 2.8%, with high certainty values for these measurements (Cu 0.99, C 0.94, Sn 0.94) (Figure 13). Copper is the main element, as bronze is mostly copper and tin. The high carbon content (6.6%) confirms the carbon layer on the bronze surface. Surface mapping, which shows the elemental distribution over a larger area, shows that the copper is uniform across the sample surface, as expected for the base material of the alloy. The carbon map shows that carbon is everywhere, as expected for a thin carbon layer. The tin map shows less presence compared to copper, but as expected for bronze. The EDS analysis confirms the carbon layer on the bronze alloy sample. The key points are: high copper (90.7%), and significant carbon (6.6%) and tin (2.8%) content, with the carbon coating applied. The surface mapping shows that carbon is uniform across the sample, and that the carbon layer is evenly applied. Copper and tin are consistent across the sample, and the bronze alloy is intact. High certainty values for the detected elements ensures the measurement is reliable, and the carbon layer is indeed present.

**Figure 14:**
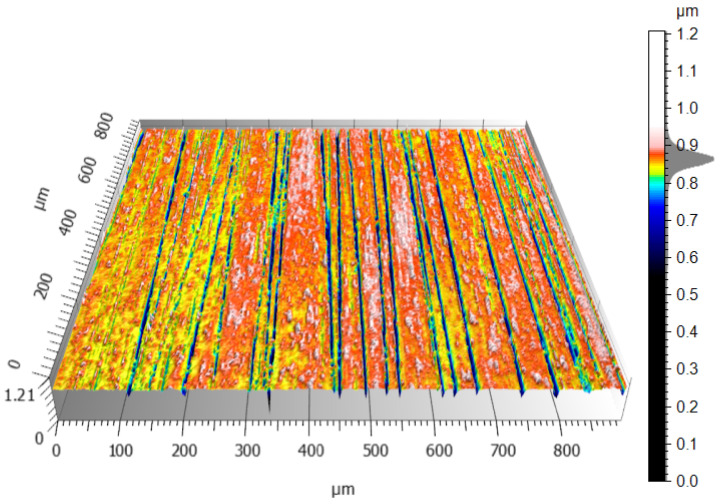
Conventionally finished tin–bronze surface.

**Figure 15:**
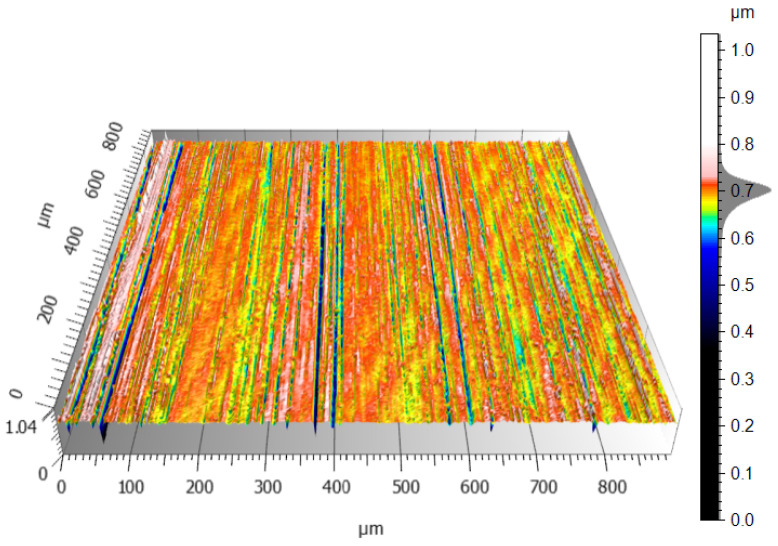
Tin–bronze surface finished with abrasive film and a carbon layer.

The CCI6000 surface roughness measurement system was used to study the surface topography of the tin–bronze samples, with and without the carbon coating. The total height of the surface without the carbon layer was about 1.2 µm (Figure 14), while the surface with the carbon layer was 1 µm (Figure 15), a difference of about 16.7%. The surface without the carbon layer is higher, the colors are distinct and varied, the height differences are more pronounced, and more scratches are deeper. The surface is less uniform, the height differences are more pronounced, and the topographical values are more dispersed. The surface with the carbon layer is lower, the colors on the profilogram are more uniform, and the height differences are smaller. The surface with the carbon layer has fewer deep scratches, which are shallower and less pronounced, and the surface is more uniform and smoother. The uniformity and smaller height differences may be due to the better distribution of the carbon layer on the alloy surface, which smoothens the surface.

**Figure 16:**
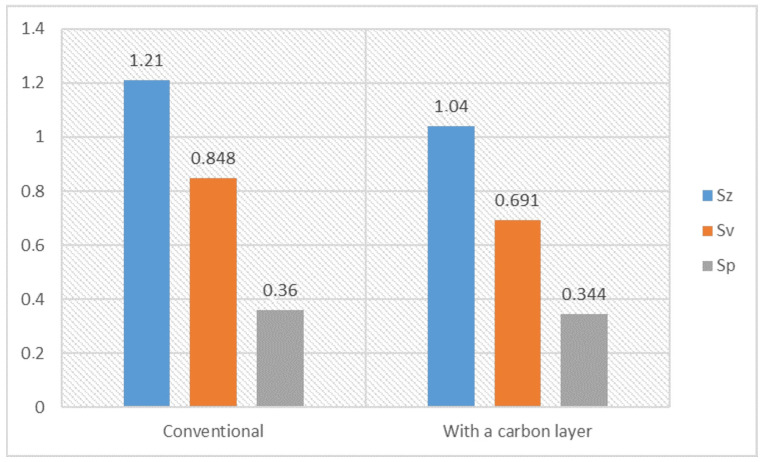
Surface roughness parameters of the bronze sample with a conventional surface and a carbon layer. The parameters measured are the maximum surface height (Sz), maximum valley depth (Sv), and maximum peak height (Sp) in µm.

Figure 16 compares the two types of samples: the conventionally finished sample and the sample with a carbon layer finished with a lapping film impregnated with graphite. The roughness parameters shown are the Sz (maximum height of the surface), Sv (maximum depth of the surface valleys), and Sp (maximum peak height of the surface). For the conventional sample, the Sz is 1.21, the Sv is 0.848, and the Sp is 0.36 µm. For the sample with a carbon layer, the Sz is 1.04, the Sv is 0.691, and the Sp is 0.344 µm. The conventional sample has a higher maximum height (Sz: 1.21 µm) than the sample with a carbon layer (Sz: 1.04); the conventional method produces a rougher surface with higher peaks. The conventional sample has a deeper maximum valley (Sv: 0.848 µm) than the sample with a carbon layer (Sv: 0.691 µm); the conventional method produces deeper valleys in the surface texture. The maximum peak height (Sp) for the conventional sample is slightly higher (Sp: 0.36 µm) than the sample with a carbon layer (Sp: 0.344 µm), the difference is smaller compared to the other parameters. The sample with a carbon layer polished with a graphite-impregnated abrasive film has lower values for all three roughness parameters (Sz, Sv, Sp) than the conventional sample. This means that the carbon layer method produces a smoother surface, with less peaks and valleys. Graphite likely contributes to finer polishing, reduces the height of the peaks and the depth of the valleys on the surface. Carbon likely accumulates in the machining marks. Finishing with a carbon layer (using graphite-impregnated abrasive film) produces a smoother surface finish than the conventional polishing method. This is evident from the lower values of the Sz, Sv, and Sp parameters in the sample with a carbon layer, and means that there is less roughness and a finer surface texture.

The conventionally finished surface, as shown in Figure 14, has higher values for roughness in terms of all the parameters. Such a surface will have more pronounced peaks and valleys; hence, it is rougher in texture. An increased Sz means that the maximum height of the surface is large, and an increased Sv means that the valleys are deeper. The more pronounced peaks increase the Sp. The general morphology indicates a less homogeneous surface, with larger irregularities and roughness. As visible from Figure 15, the roughness values for the surface finished with a carbon layer are less. The application of a carbon layer makes the surface smoother, with fewer and less sharp peaks and valleys. The reduced Sz value indicates a lower maximum height, while the shallower valleys are indicated by a lower Sv value. Noticeably, the Sp value decreases, which means the peaks are less pronounced. The carbon layer helps to create a more uniform and smoother surfaces, evidenced by the decrease in the roughness parameters. The carbon content mapping analysis across the entire surface showed the presence of this element. However, by analyzing the Sv roughness parameter, it can be assumed that a significant portion of the carbon on the surface accumulates in the valleys of the machining marks. This is a very beneficial phenomenon that positively affects the interacting elements. A similar concept is employed when honing cylinders, where an array of machining marks is actually induced for providing lubricants with a conduit into these valleys, promoting good operational performance. The idea here is that the lubricant is fed in during machining; therefore, there is no additional need for access, as far as refueling with lubricant is concerned.

**Figure 17:**
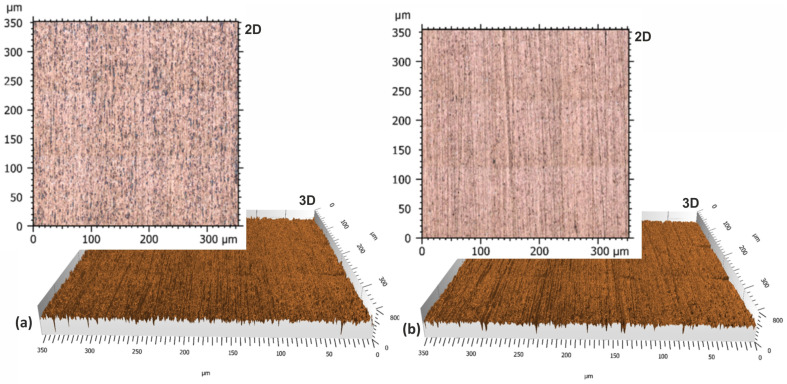
Confocal microscope images in optical mode, showing the true colors of the bronze alloy surface sample after the lapping process: (**a**) conventional finish and (**b**) finish with abrasive films and a carbon layer.

Figure 17 shows two optical images taken with an OLS 4000 confocal microscope. There is no color difference between the two surfaces, which means that the carbon layer is transparent. A layer is considered transparent when its thickness is small enough to prevent significant absorption, or the scattering of visible light, which ranges in terms of the wavelength from about 400 to 700 nanometers.

## 4. Summary and Conclusions

This paper deals with the creation of a carbon layer on workpiece surfaces using graphited-impregnated lapping films. The process consists of impregnating the lapping film with graphite, supplying it using a controlled speed by the system, and pressing it onto the rotating workpiece surface. Such graphite in the machining zone, combined with pressure, provides an even graphite distribution, and results in a shiny surface that has a thin carbon layer. The experimental setup was conducted using the GW1 micro-finishing attachment, which controlled the tool feed rates, oscillation frequencies, and pressure forces. The surface topography measurements were conducted using the Talysurf CCI 6000 system and SEM and ED analysis for verification of the presence and uniformity of the carbon layer. The surface properties and roughness characteristics show massive improvements in the soda–lime glass and CuSn7Zn4Pb6/RG7 tin–bronze alloy. The GDOES analysis also confirmed the carbon layer deposition and its consequences in terms of the elemental composition on the treated surfaces. Among the many scientific merits of the work is the novel integration of micro-finishing with carbon layer deposition in a single process, which truly represents another step forward in the development of surface engineering. Two key contributions of the new technique are achieved, namely surface smoothness and the improvement of the tribological properties; therefore, it is a very valuable tool for a wide range of industrial applications. It points to new methods of surface treatment that can change the way surfaces are finished across many industries, where the conventional reduction of friction is not possible. The findings in this study present a solid basis that can be used for further research and development in the field of advanced surface coatings and their applications.

- The microabrasive rolls surface finishing process is combined with the thin carbon layering process. In this way, it is possible to obtain a carbon texture, while reducing the roughness of the machined surface. Both these characteristics are of great tribological importance.
- The lapping process and the application of a carbon layer reduces surface roughness and increases its uniformity. The most significant differences in the surface roughness parameters were noted for the Sv parameter on surfaces smoothed with graphite-impregnated abrasive film, which concerns the maximum valley depth. The Sv parameter significantly decreased in all the conducted studies, suggesting that the main accumulation sites for carbon on the machined surface are the valleys of the machining marks. Additionally, the cross-hatching of these marks, a characteristic feature of the micro-finishing process, provides an additional benefit for the subsequent interaction of the finished surface with other elements.
- The SEM and EDS and GDOES studies confirm the successful deposition of a carbon film on the machined surfaces. These investigations have shown that on the machined surface, there exists a contribution by carbon for both materials that did not contain C initially.
- The integrated method of applying a thin carbon film in the form of texture and the micro-smoothing surface process shows the potential for applications requiring a higher surface finish and wear resistance. Therefore, further research in the field of coating properties is recommended.

## 5. Patents

W. Kacalak, K. Tandecka, B. Bałasz, K. Rokosz: Method for Producing Carbon Nanolayers on Surfaces at the Time of Micro-Smoothing Them with Abrasive Films. Patent No. Pat. 240472, filed on 17 April 2018, granted on 17 January 2022, valid until 17 April 2038. Application No. P.425253. IPC Classification: B82Y 30/00, B24D 11/02, B24D 3/00, B32B 7/10.

## Figures and Tables

**Table 1:** Experimental machining conditions.

Workpiece Material	Pressure Roll Hardness	Pressure Force	Tool Speed	Workpiece Speed	Oscillation Frequency	Processing Time
Tin–bronze alloy (CuSn7Zn4Pb6/RG7) Soda–lime glass	50^0^Sh	50 N	160 mm/min	10 m/min	80 Hz	60 s

## Data Availability

The data are contained within the article.
